# COVID-19 preventive behavior among university students in Southeast Asia: Effects of knowledge, concern, awareness, and perceived risk

**DOI:** 10.3389/fpubh.2022.958021

**Published:** 2022-09-09

**Authors:** Abdullah Al Mamun, Naeem Hayat, Huynh Thi My Dieu, Noor R. Zainol, Anas A. Salameh

**Affiliations:** ^1^UKM - Graduate School of Business, Universiti Kebangsaan Malaysia, Bangi, Malaysia; ^2^Global Entrepreneurship Research and Innovation Centre, Universiti Malaysia Kelantan, Kota Bharu, Malaysia; ^3^UFM Graduate School, University of Finance and Marketing, Ho Chi Minh City, Vietnam; ^4^College of Business Administration, Prince Sattam Bin Abdulaziz University, Al-Kharj, Saudi Arabia

**Keywords:** global health, pandemic, coronavirus disease 2019, multiple group analysis, preventive behavior

## Abstract

The persistent rise of pandemics across the globe in recent times has led to the prescription of several collaborative preventive strategies to reduce the effect that the pandemic has on public health. Consistent monitoring and surveillance appear to be the only available approach to detecting and classifying the issues of public health threats. Global pandemic threats demand public co-operation to take preventive actions at a personal level so that the risk of infectious diseases can be contained. Said that, this study explored the influence of awareness of precaution measures (APM), concerns about coronavirus disease 2019 (COVID-19) (CAC), knowledge of COVID-19 (KOC), and perceived risk (PRK) on preventive behavior (PRB), as well as the effect of age and gender on the relationships among the studied variables. Quantitative data were collected from 551 university students across Malaysia and Vietnam through field survey and online survey, respectively. The data collection was performed from 13 March to 23 March 2020. Partial least square structural equation modeling (PLS-SEM) was employed for data analysis. The multiple group analysis (MGA) technique was applied to compare the data retrieved from the respondents based on age and gender. The results revealed that APM, CAC, KOC, and PRK on PRB significantly influenced PRB toward COVID-19. In light of the two personal factors, age and gender, significant variances were noted for age and KOC, while PRK on PRB on the PRB toward COVID-19. Based on the study outcomes, APM emerged as the most significant predictor of PRB, followed by PRK on PRB, and CAC. Since a large fraction of the world reside in rural areas and have high-level interaction with animals, the provision of education at all level can harness the attitude to adopt PRB toward COVID-19. As such, policymakers need to work with the young generation so that the latter may serve as change agents to spread the message of taking precautions and adopting effective PRB toward COVID-19.

## Background

The emergence of new infectious diseases is on the rise. In the past two decades, severe acute respiratory syndrome (SARS), triple reassortant H1N1 influenza, and Middle East respiratory syndrome coronavirus (MERS-COV) have resulted in substantial economic and human life loss (1). The global spread of these health threats emphasizes the existing vulnerabilities that lead to the lockdown of cities, deteriorating global trade, and travel bans (2). This outbreak has not only resulted in agonizing death toll but also caused a series of social and psychological reactions, with unpredictable consequences for society (3). This disaster's side effects have caused many challenges to people's lives and mental health worldwide (4). The emergence of unique viruses is inherently unpredictable, as it is impossible to predict the emergence of any pandemic before its first occurrence. Nonetheless, continuous surveillance is an effective strategy to recognize the development of infectious diseases (5). More than 70% of infectious diseases derive from animals. Human, animals, and environmental interactions are critical in comprehending the emergence of infectious diseases (6). Nevertheless, intense reconnaissance is the only available strategy to identify and recognize the ecology, evolution, and transmission of potential infectious diseases (7).

The origin and spread of infectious diseases offer the following three relevant patterns. The frequency of infectious diseases is on the rise, and it requires a consistent and global coordinated effort to enable the surveillance of infectious diseases (1). Infectious diseases are high in highly populated areas. An adverse climate change, altered human relationships with nature, and escalated human travel are some known causes of a pandemic (7). The high interaction between humans and wildlife increases the risk of pandemic emergence (2). The three stages of disease emergence are pre-emergence, localized emergence, and full pandemic emergence (6).

The spread of infectious diseases broadly rests on the host physiology attributes of age and immunology competence (2). The human social aspect has mostly missed exploring the spread and prevention of a pandemic. Understanding the processes underlying self-protection decisions is essential for effective risk communication during the COVID-19 pandemic (8). Implementing most measures based on individual behavior change can be challenging (8). The pre-emptive approaches have been termed the only available strategy to control the spread of infectious diseases and the general threat to public health (7). Pre-emptive strategies can significantly reduce the impact of the pandemic and reoccurring of the new version of COVID-19, while human personal and social aspects are significant in promoting preventive behavior (PRB) (5). Nevertheless, human vulnerabilities stress taking systematic and pre-emptive approaches to halt the spread of global threats and reduce the emergence of the pandemic (6).

The coronavirus disease 2019 (COVID-19) disease pandemic had spread across the world. Globally, as of 29 July 2022, there have been 572,239,451 confirmed cases of COVID-19, including 6,390,401 deaths (9). The South Eastern Asian countries also experienced the spread of COVID-19 and lock down implemented to reduce the spread of the COVID-19 (2). The initial public perception of COVID-19 based on fear and pre-emptive actions rested on public preparedness. Lockdown is not a permanent and welcoming strategy to control the spread of COVID-19 (7). Public preparedness reflects the awareness of the precautionary measures to reduce the effect of COVID-19 (11). Old age individuals and women appear to be more inclined to take preventive actions against COVID-19 (10). The recent figures depict that 4.7 million individual were infected in Malaysia and the death toll reached 16,000, whereas the 10.8 million people were infected by COVID-19 in Vietnam and death toll reached 43,000 (9).

COVID-19 is not entirely controlled in the South East Asian countries, and the new version of COVID-19 still reminds us that the best possible way to deal with COVID-19 is taking pre-emptive strategies. Nevertheless, how individuals form pre-emptive behavior toward COVID-19 is yet to be explored. Moreover, understanding the motivations for preventive behaviors is critical to increasing compliance and improving the effectiveness of containment measures through adequate health campaigns (4).

It is important to understand public behavior to develop effective communication strategies and ensure high compliance with protective practices (3). This study investigated the influence of awareness of precaution measures (APM), concern about COVID-19 (CAC), knowledge of COVID-19 (KOC), and perceived risk (PRK) on PRB toward COVID-19. In addition, this study looked into the impact of two personal attributes (age and gender) on the relationships of APM, CAC, KOC, and PRK of COVID-19 with PRB toward COVID-19.

The remaining part of the article is composed of the following: the next section presents the relevant literature on individual PRB influenced by APM, CAC, KOC, and PRK of COVID-19. In addition, the moderating roles of age and gender on the COVID-19 PRB are discussed. Section Methods describes the methodology applied in this study based on the literature review and presents the formulated hypotheses. The analysis and the results are reported in Sections Results and Discussion, respectively. Lastly, Section Conclusion concludes the study, offers the path of future direction, and reports the study limitation. To control the spread of COVID-19, developing public health campaigns to promote preventive behaviors seems to be a critical method. To do this, it is necessary to understand the psychosocial determinants of COVID-19 prevention behaviors. Therefore, findings from this research could inform communication campaigns and other behavior change efforts to reduce the spread of this epidemic.

## Literature review

### Preventive behavior

Population co-operation and taking appropriate preventive actions are necessary to hinder the spread of infectious diseases (6). Preventive actions include reducing public places' use, how to prevent cough, intensive hand washing, surface disinfection, and talking with everyone about how to forbid (12). Public engagement to cooperate and to start taking precautions for preventing infectious diseases is essential (13). The PRB measures are the only effective strategies to minimize the infection rate (5). The imperative approach is to prevent physical contact among individuals, as this approach was reported to reduce 23% of influenza diffusion (14). Hand washing and wearing face masks were also significant in combatting SARS and MERS-COV (15).

### Awareness of precaution measures

Prevention measure denotes a valuable tool that halts the spread of infectious diseases (14). Prevention measures suggest the guidelines of activities to prevent the expanse of infectious diseases (6). Enhancing the APM is the most cost-effective strategy to control the harmful effect of the diseases (13). Launching social alertness programs *via* conventional and social media has enhanced APM (10). Awareness is the best tool to inform the population about the precautionary daily routines to minimize the harmful impact of infectious diseases (1). People's awareness of COVID-19 affects their perceived risk and effectiveness in disease prevention (4). The perceived threat of the COVID-19 pandemic plays an essential role in estimating people's awareness of disease severity and willingness to adhere to preventive behaviors (16). The aspect of APM amidst the population empowers the practice of PRB at the individual level in a true sense (10). In the intensively difficult situation of spreading infectious diseases at an alarming rate, the best strategy is to create awareness to inform and promote PRB in the population (7). Hence, the following is hypothesized:

Hypothesis 1 (H1): *Awareness of precaution measures for COVID-19 has a significantly positive effect on the preventive behavior toward COVID-19*.

### Concern for COVID-19

Concern about pandemics refers to the realization of an infectious disease's death to the general public (5). Assessing the gravity of the situation directs the public response toward the assessment of the situation and the likelihood of adopting the proposed PRB (17). The level of concern has been positively linked with PRB among respondents from the Kingdom of Arabia toward MERS (7). A person with a high social concern is willing to sacrifice their own desires when they think it will harm others (18). Awareness of COVID-19 affects risk awareness and disease prevention effectiveness (4). The implementation of lockdown both endorses and greatly enhances CAC (10). Thus, this study proposes the following:

Hypothesis 2 (H2): *Concern about COVID-19 has a significantly positive effect on the preventive behavior toward COVID-19*.

### Knowledge of COVID-19

Knowledge is beyond awareness about infectious diseases. Knowledge describes the common symptoms in identifying an infectious disease (2). Knowledge is a people's awareness of their physical and mental state, understanding of COVID-19, those susceptible to COVID-19, and their awareness of the risk of infection (12). Knowledge entails the form of behavior, action, or checklist that enables one to identify the presence of the disease (13). Lack of knowledge involves misunderstanding or the inability to isolate the distinguishable signs that describe the presence of an infectious disease (5). Knowledge has an integral role in the execution of the attitude to avoid infectious diseases (2). Consistent knowledge of infectious diseases enhances the concern and the behavior to take precautionary actions to avoid the disease (10). Past studies support the notion that knowledge about infectious diseases leads to the exhibition of preventative behavior (7). Knowledge is related to practicing preventive measures (12), and limited knowledge leads to wrong perceived risks (18). Therefore, the following hypothesis is prescribed:

Hypothesis 3 (H3): K*nowledge of COVID-19 has a significantly positive effect on the preventive behavior toward COVID-19*.

### Perceived risk

Perception of risk is one's personal understating of a negative likelihood that may occur. Risk is a natural human instinct as an assessment of the surrounding environment about the likelihood of a negative outcome (1). People tend to adopt precautionary actions for the high-risk perception of a disease or vice versa (7). The perception of risk is one's personal understanding and varies between individuals (19), encouraging people to engage in protective behavior to reduce the potential risk (20). The risk associated with getting an infectious disease is highly linked to adopting PRB to avoid the disease (13). Risk perception can predict significant preventive behavior (21). Nevertheless, both PRK and APM were found to influence PRB (10) interactively. Hence, the following hypothesis was forwarded:

Hypothesis 4(H4): *Perceived risk significantly positively affects the preventive behavior toward COVID-19*.

### The effects of age and gender

Systemic problems can influence preventive behaviors that increase the vulnerability of demographic subgroups, especially racial and ethnic minorities (22). Behavioral attitude greatly varies based on the personal factors of an individual (10). It was reported that women displayed higher vulnerability than men for H1N1 influenza among the Korean sample (6). Soltan et al. (12) revealed that female students have more knowledge and practice preventive behaviors than male students. The impact of one's age and gender can significantly vary the fundamental correlation between attitude and behavior toward pandemics in Saudi Arabia (7). One's age develops a particular life role and advances, taking more responsible actions to avoid risk or engage in PRB toward infectious diseases (10). Similarly, gender has been linked with certain roles and preferences that can lead to specific attitudes (7). Jang et al. (6) posited that gender and age had influenced the PRB among university students in Malaysia and Vietnam. Hence, it is imminent to explore the effect of age and gender on PRB toward COVID-19, as postulated in this study. As such, the following are hypothesized:

*H*_1*MGA*_*: The respondents' age moderates the relationships of awareness of precautionary measures for COVID-19, concern about COVID-19, knowledge of COVID-19, and perceived risk with preventative behavior toward COVID-19; such as relationship is stronger for the older respondents than the young to engage in preventative behavior toward COVID-19*.

*H*_2*MGA*_*: The respondents' gender moderates the relationships of awareness of precautionary measures for COVID-19, concern about COVID-19, knowledge of COVID-19, and perceived risk with preventative behavior toward COVID-19; such as relationship is stronger for the men respondents than the female to engage in preventative behavior toward COVID-19*.

## Methods

### Data collection and sample selection

This study selected two universities, one from Malaysia (Universiti Malaysia Kelantan) and one from Vietnam (University of Finance—Marketing). The required sample size for the study, estimated using G-Power 3.1 with a power of 0.95, the effect size of 0.15, and four predictors, was 74 (G^*^Power Source: https://webpower.psychstat.org/models/kurtosis/). As required in PLS-SEM, the minimum threshold was 100 samples (23). Therefore, 500 respondents from Malaysia and Vietnam were involved in the data collection process for this study to hinder any potential complications stemming from the small sample size.

In Malaysia, data were collected from students after lecture sessions selected randomly. Data were collected during the lecture from everyone attending the selected lecture. As the data collected during the lecture, the response rate in Malaysia was 100%. Moreover, in Malaysia, nearly 60% of the students in public universities are female; therefore, high proportion of respondents expected to be female. Meanwhile, the university was already closed in Vietnam due to the COVID-19 outbreak; the data were collected *via* an online survey. The students were encouraged to share and link the message posted on several online platforms (including Facebook groups, students' Facebook pages, and Instagram) to as many students as possible to increase visibility. From Vietnam, as the data were collected using a google form, this study cannot confirm the number of students who received the request, therefore cannot confirm the response rate. However, the study online data collection was performed according to the checklist for reporting results of internet E-Surveys (CHERRIES) guidelines. As a result, the data were gathered from 551 students (245 from Malaysia and 306 from Vietnam) from 13th to 23rd March 2020.

### Research instrument

This study adopted a premeditated survey and exploited several previously validated scales. All items used in this study presented in Table 1. The questionnaire used the five-point Likert scale that ranged from 1 = strongly disagree to 5 = strongly agree for each construct. Complete data are provided as Addition File (Cov Data.CSV).

**Table 1:** Survey instrument.

**Variable and items**	**References**
**Knowledge about COVID-1**	
Common signs of infection include:	WHO (24)
Respiratory symptoms, fever, cough, shortness of breath, pneumonia, SARS, death	
**Concern about COVID-1**	
I avoid leaving my home nowadays	Almutairi et al. (7)
If 1 decide to travel, COVID-19 may prevent me from traveling	
The government should restrict travel from and to the areas of the disease to avoid spread of disease	
The government should isolate infected patients in special hospitals	
The government should monitor new arrivals from other countries	
The government must be ready to close schools, colleges, and universities if the number of cases increases	
**Awareness of precautionary measure**	
I need to wash my hands frequently	WHO (24)
I need to maintain social distancing	
I need to avoid touching my eyes, nose, and mouth	
I need to practice respiratory hygiene	
If I have fever, cough, and difficulty in breathing, I must seek medical care as early as possible	
I need to stay informed and heed the advice given by the healthcare provider	
I need to cover my nose and mouth with a tissue when coughing or sneezing	
I need to throw the tissue into the trash after using it	
I need to use a face mask to cover my nose and mouth in crowded places	
If I have flu symptoms, I need to avoid normal activities such as going to work, school, travel, shopping, etc.	
**Perceived risk**	
In your opinion, what is the likelihood that COVID-19 will reach your community?	Ibuka et al. (19)
In your opinion, what is the likelihood that you will personally encounter someone infected with COVID-19?	
Do you think a large proportion of Malaysians/Vietnamese will suffer from COVID-19 during this outbreak?	
Do you think a large proportion of people worldwide will suffer from COVID-19 during this outbreak?	
**Engagement in precautionary activities—Preventive behavior**	
I wash my hands more often than usual because of COVID-19	Parmeggiani et al. (25)
I wear a face mask because of COVID-19	
I avoid or reduce outdoor activities or attend meetings this week because of COVID-19	
I avoid or reduce using public transportation such as the bus or the subway this week because of COVID-19	
I avoid or reduce using healthcare facilities such as hospitals or public health centers this week because of COVID-19	
I avoid or reduce visiting crowded markets, department stores, or large discount stores this week because of COVID-19	
I am following the television or radio news more closely in response to the COVID-19 outbreak	
I have searched the internet for additional information about the COVID-19 outbreak	
I have canceled or changed travel plans in response to the COVID-19 outbreak	
I stay home from school in response to the COVID-19 outbreak	
I stay home from work in response to the COVID-19 outbreak	
I have canceled or changed social plans in response to the COVID-19 outbreak	

### Common method variance

Cross-sectional studies are commonly associated with the issue of CMV; assessing and correcting CMV can be performed using multiple methodological and statistical tools (26). In this study, Harman's one-factor test was applied to determine the effect of CMV as a diagnostic technique (26). The single factor accounted for 42.55%, which is below the recommended threshold of 50% in Harman's one-factor test, thus approving the inconsequential influence of CMV on this study. In addition, to establish the strength of the CMV evaluation, the correlations among the study latent constructs were estimated, wherein a correlation that scores below 0.9 signifies the absence of CMV (26). This study satisfied this requirement as well. Furthermore, this study evaluated the common method variance by following Kock's (27) recommendation to test the full collinearity of all the constructs. All the study constructs were regressed on the common variable, and the variance inflation factor (VIF) values are <3.3 indicating the absence of bias from the single-source data. Full collinearity analysis shows no issue of single-source bias (see Table 2).

**Table 2:** Full collinearity test.

**APM**	**CAC**	**KOC**	**PRK**	**PRB**
1.004	1.707	1.859	1.402	1.237

APM, awareness of precaution measurement; CAC, concern about COVID-19; KOC, knowledge of COVID-19; PRK, perceived risk; PRB, preventative behavior.

### Multivariate normality

Multivariate normality for the study data was assessed with the Web Power online tool, which utilizes the R function discussed in the previous section to obtain skewness and kurtosis on a Web server and produces the same results as SAS, SPSS, and R [(28); pp. 1731]. The use of multivariate analysis helps to establish the robustness of the analysis and adds to the transparency of the data presentation (29). The calculated Mardia's multivariate skewness and kurtosis coefficient, as well as *p*-values, displayed that the study data had a non-normality issue as the *p*-values were below 0.05 (29).

### Data analysis method

The study model was investigated using the partial least square structural equation modeling (PLS-SEM) *via* Smart-PLS software 3.1. Smart PLS is a multivariate analysis instrument that appraises path models with composite-based latent constructs (29). The PLS-SEM is commonly associated with small datasets and addresses complex models with composites with no assumption of goodness-of-fit estimation compared to covariance-based SEM (30). The two-stage approach is recommended by using the Smart PLS 3.1 (31). We utilize the SmartPLS to have the individual parameter's significance and provide the statistical inference and sign change correction. Our dataset has non-normality, and the nature of the study is explorative. Data analysis and the first model measurement were performed on the model to test the reliability and validity of the study constructs (29). Next, the second stage involved assessment of the structural model correlations and hypotheses testing with significance levels achieved *via* bootstrapping. Model estimation was performed with *r*^2^, *Q*^2^, and effect size of *f*^2^ that describe the path effect from exogenous construct to endogenous construct (29). The multiple group analysis (MGA) in PLS-SEM enables scholars to distinguish the variances in pre-defined groups under investigation (32). The MGA refers to a dexterous method that detects the differences between the groups within a dataset (21). The MGA assists scholars to appraise the changes found in the structural paths of the various groups that subsist in the data (32). In this study, the first step was to generate groups based on the categorical variables of interest, including age, gender, or income. Next, the path coefficients of the groups were analyzed to determine if the two groups are significantly diverse from each other based on the guidelines proposed by Henseler et al. (32). The differences that exist in the dataset are based on the characteristics of the samples, which may not be evident in aggregated data, and path coefficients of the group data reflect the statistical variance by using MGA, to establish the statistically significant variances in data based on categorical bases (32).

## Results

### Demographic characteristics

Data were collected from Malaysia and Vietnam. As tabulated in Tables 3, 55.5% of the respondents were from Vietnam, while the rest were from Malaysia. The majority of the respondents were females, at 69.5% of the total respondents. As for age, 14% of the respondents were 20 or <20 years old, while 75.7% of the respondents were between 21 and 30 years of age. Respondents in the 31–40 age range were 6.4%, whereas 2.7% of the respondents belonged to the 41–50 age range, and the remaining were above 51 years old. Most respondents were single at 91.1%, while the remaining were married.

**Table 3:** Profile of the respondents.

	***n***	**%**		***n***	**%**
**Gender**			**Age (years)**		
Male	167	30.3	20 and below	81	14.7
Female	383	69.5	21–30	417	75.7
Total	550	100	31–40	35	6.4
			41–50	15	2.7
			51 and above	3	0.5
			Total	551	100
**Marital status**					
Single	502	91.1	**Country**		
Married	46	8.3	Malaysia	245	44.5
Others	3	0.5	Vietnam	306	55.5
*Total*	551	100	Total	551	100

### Descriptive statistics

Table 4 presents the results pertaining to KOC. It was found that most students were more aware of the COVID-19 common symptoms, including fever and cough, than those of pneumonia and SARS.

**Table 4:** Knowledge of COVID-19.

**Common signs of infection include**	**Strongly disagree**	**Disagree**	**Neither agree nor disagree**	**Agree**	**Strongly agree**
Respiratory symptoms	14 (2.5%)	15 (2.7%)	73 (13.2%)	261 (47.4%)	188 (34.1%)
Fever	13 (2.4%)	1 (0.2%)	13 (2.4%)	258 (46.8%)	266 (48.3%)
Cough	13 (2.4%)	0 (0%)	18 (3.3%)	242 (43.9%)	278 (50.5%)
Shortness of breath	18 (3.3%)	46 (8.3%)	75 (13.6%)	201 (36.5%)	211 (38.3%)
Pneumonia	13 (2.4%)	25 (4.5%)	135 (24.5%)	210 (38.1%)	168 (30.5%)
Severe acute respiratory syndrome	16 (2.9%)	21 (3.8%)	103 (18.7%)	274 (49.7%)	137 (24.9%)
Death	30 (5.4%)	30 (5.4%)	56 (10.2%)	204 (37.0%)	231 (41.9%)

In light of the rising CAC (see Table 5), most of the students strongly believed that the government should restrict travel from and to the areas of the disease to avoid the spread of disease, isolate infected patients in special hospitals, and monitor new arrivals from other countries.

**Table 5:** Concerns about COVID-19.

	**Strongly disagree**	**Disagree**	**Neither agree** **nor disagree**	**Agree**	**Strongly agree**
I avoid leaving my home nowadays	7 (1.3%)	11 (2.0%)	105 (19.1%)	258 (46.8%)	170 (30.9%)
If I decide to travel, COVID-19 may prevent me from traveling	24 (4.4%)	18 (3.3%)	36 (6.5%)	231 (41.9%)	242 (43.9%)
The government should restrict travel from and to the areas of the	17 (3.1%)	3 (0.5%)	27 (4.9%)	212 (38.5%)	292 (53%)
disease to avoid the spread of the disease					
The government should isolate infected patients in special hospitals	11 (2%)	0 (0%)	22 (4%)	214 (38.8%)	304 (55.2%)
The government should monitor the new arrivals from other countries	10 (1.8%)	1 (0.2%)	17 (3.1%)	207 (37.6%)	316 (57.4%)
The government must be ready to close schools, college and universities	10 (1.8%)	4 (0.7%)	19 (3.4%)	173 (31.4%)	345 (62.6%)
if the number of cases increases					

Table 6 presents the results for APM. It was observed that most students knew they needed to wash their hands frequently and avoid touching their eyes, nose, and mouth. They reckoned that they needed to seek medical aid as early as possible if they had a fever, cough, and difficulty breathing. They also related to the importance of being informed and heeding the advice given by healthcare providers. They know they must use a facemask to cover their nose and mouth in crowded places. If they have flu symptoms, they know they must avoid normal activities, such as going to work, school, travel, and shopping.

**Table 6:** Awareness of precautionary measures.

	**Strongly disagree**	**Disagree**	**Neither agree nor disagree**	**Agree**	**Strongly agree**
I need to wash my hands frequently	9 (1.6%)	1 (0.2%)	5 (0.9%)	153 (27.8%)	383 (69.5%)
I need to maintain social distancing	10 (1.8%)	7 (1.3%)	52 (9.4%)	225 (40.8%)	257 (46.6%)
I need to avoid touching eyes, nose and mouth	9 (1.6%)	2 (0.4%)	36 (6.5%)	201 (36.5%)	303 (55%)
I need to practice respiratory hygiene	9 (1.6%)	4 (0.7%)	47 (8.5%)	227 (41.2%)	264 (47.9%)
If I have fever, cough and difficulty breathing, seek medical care early	8 (1.5%)	3 (0.5%)	19 (3.4%)	192 (34.8%)	329 (59.7%)
I need to stay informed and follow advice given by our healthcare provider	8 (1.5%)	1 (0.2%)	13 (2.4%)	186 (33.8%)	343 (62.3%)
I need to cover my nose and mouth with a tissue when coughing or sneezing	9 (1.6%)	0 (0%)	14 (2.5%)	172 (31.2%)	356 (64.6%)
I need to throw the tissue in the trash after I use it	9 (1.6%)	2 (0.4%)	21 (3.8%)	272 (31.2%)	374 (63%)
I need to use face mask to cover my nose and mouth in crowded places	8 (1.5%)	3 (0.5%)	15 (2.7%)	174 (31.6%)	351 (63.7%)
If I have flu symptoms appeared, I need to avoid normal activities such as going to work for school, travel, shopping, etc.	11 (2.0%)	5 (0.9%)	20 (3.6%)	185 (33.6%)	330 (59.9%)

Table 7 tabulates the findings related to PRK. Most students were aware of the likelihood that COVID-19 can reach their community, and one can get infected by personally encountering a COVID-19-infected patient. Many students were aware of the possibility of a large-scale outbreak in their country and worldwide.

**Table 7:** Perceived risk.

	**Definitely not**	**Probably not**	**Possibly**	**Probably**	**Definitely**
In your opinion, what is the likelihood that COVID-19 will reach your community?	8 (1.5%)	38 (6.9%)	97 (17.6%)	247 (44.8%)	161 (29.2%)
In your opinion, what is the likelihood that you will personally encounter somebody infected with COVID-19?	19 (3.4%)	54 (9.8%)	113 (20.5%)	249 (45.2%)	116 (21.1%)
Do you think a large proportion of Malaysian/Vietnamese will suffer from COVID-19 during this outbreak?	19 (3.4%)	60 (10.9%)	151 (27.4%)	219 (39.7%)	102 (18.5%)
Do you think a large proportion of people worldwide will suffer from COVID-19 during this outbreak?	13 (2.4%)	24 (4.4%)	69 (12.5%)	289 (52.5%)	156 (28.3%)

Table 8 displays the results for engagement in precautionary activities. Most students washed their hands more often than usual due to the COVID-19 outbreak. Nearly 84% of the students reported wearing face masks because of COVID-19. More than 86% of the students confirmed that they avoided or reduced outdoor activities or attending meetings because of COVID-19. Besides, more than 85% confirmed that they avoided or reduced using healthcare facilities, such as hospitals and public health centers, due to the COVID-19 outbreak.

**Table 8:** Engagement in precautionary activities—preventive behavior.

	**Strongly disagree**	**Disagree**	**Neither agree nor disagree**	**Agree**	**Strongly agree**
I wash my hands more often than usual because of COVID-19	8 (1.5%)	2 (0.4%)	23 (4.2%)	215 (39%)	303 (55%)
I wore a face mask because of COVID-19	9 (1.6%)	11 (2%)	69 (12.5%)	218 (39.6%)	244 (44.3%)
I avoid or reduced outdoor activities or attending meetings this week because of COVID-19	8 (1.5%)	3 (0.5%)	59 (10.7%)	232 (42.1%)	249 (45.2%)
I avoid or reduced using public transportation such as the bus or the subway this week because of COVID-19	9 (1.6%)	4 (0.7%)	65 (11.8%)	239 (43.4%)	234 (42.5%)
I avoid or reduced using healthcare facilities such as hospitals or public health centers this week because of COVID-19	14 (2.5%)	26 (4.7%)	140 (25.4%)	214 (38.3%)	157 (28.5%)
I avoid or reduced visiting crowded markets, department stores, or large discount stores this week because of COVID-19	8 (1.5%)	8 (1.5%)	81 (14.7%)	239 (43.4%)	215 (39%)
I am following television or radio news more closely in response to the COVID-19 outbreak	8 (1.5%)	4 (0.7%)	38 (6.9%)	215 (39%)	286 (51.9%)
I have searched the internet for additional information on the COVID-19 outbreak	8 (1.5%)	3 (0.5%)	43 (7.8%)	273 (43%)	260 (47.2%)
I have canceled or changed travel plans in response to the COVID-19 outbreak	8 (1.5%)	3 (0.5%)	49 (8.9%)	223 (40.5%)	268 (48.6%)
Me and/or my children(s) stayed home from school in response to the COVID-19 outbreak	14 (2.0%)	3 (0.5%)	72 (13.1%)	214 (38.3%)	247 (44.8%)
I have stayed home from work in response to the COVID-19 outbreak	29 (5.3%)	60 (10.9%)	95 (17.2%)	183 (33.2%)	184 (33.4%)
I have canceled or changed social plans in response to the COVID-19 outbreak	15 (2.7%)	5 (0.9%)	76 (13.8%)	204 (37%)	251 (45.6%)

### Validity and reliability

Following the endorsement of Hair et al. (29), reliability for the latent constructs of this study was determined with Cronbach's alpha (α), DG rho, and composite reliability. The values of Cronbach's alpha for all the constructs were above the threshold of 0.70, and the minimum Cronbach's alpha value was 0.835 (33). The results are tabulated in Table 9. Next, all DG rho values exceeded the threshold of 0.70, and its minimum value was 0.845 (29). The composite reliability values also were above the threshold of 0.70, where the minimum value of CR was 0.888 (33). These results signify that the latent constructs had achieved adequate reliability and thus were fit for further analyzed. The average variance extracted (AVE) for all items must exceed 0.50 to attain the acceptable convergent validity to support the uni-dimensionality of each construct (29). The items displayed that the constructs possessed acceptable convergent validity (see Table 9). Both item loading and cross-loading indicate the discriminant validity of the constructs.

**Table 9:** Reliability analysis.

**Variables**	**No. of** **items**	**Cronbach's** **alpha**	**DG** **rho**	**Composite** **reliability**	**Average variance** **extracted**	**Variance inflation** **factor**
Awareness of precautionary measures	7	0.942	0.934	0.951	0.659	2.650
Concern about COVID-19	6	0.852	0.867	0.892	0.584	2.522
Knowledge of COVID-19	10	0.851	0.868	0.888	0.535	1.903
Perceived risk	4	0.835	0.845	0.889	0.667	1.247
Preventive behavior	12	0.927	0.935	0.937	0.557	–

All the study constructs exhibited fitting discriminant validity (see Table 10). The Fornell–Larcker criterion and heterotrait–monotrait (HTMT) ratio were employed to determine the discriminant validity of the study constructs. The Fornell–Larcker criterion is estimated with the square root of the respective construct AVE. The square root of AVE for the construct must exceed the correlation among the other constructs (29). The HTMT ratio should be <0.90 to establish discriminant validity for the study constructs (33). The VIF values below 3.3 display nil multicollinearity issues (31). Table 9 shows that the study confirmed each construct's discriminant validity.

**Table 10:** Outer loading and cross loadings.

	***APM***	**CAC**	**KOC**	**PRK**	**PRB**
APM—Item 1	*0.773*	0.471	0.455	0.257	0.395
APM—Item 2	*0.844*	0.600	0.613	0.263	0.535
APM—Item 3	*0.834*	0.612	0.624	0.272	0.538
APM—Item 4	*0.654*	0.355	0.410	0.402	0.387
APM—Item 5	*0.654*	0.375	0.346	0.100	0.383
APM—Item 6	*0.752*	0.398	0.413	0.192	0.407
APM—Item 7	*0.562*	0.330	0.391	0.374	0.370
CAC—Item 1	0.376	*0.560*	0.419	0.285	0.460
CAC—Item 2	0.360	*0.675*	0.418	0.229	0.397
CAC—Item 3	0.458	*0.790*	0.545	0.263	0.473
CAC—Item 4	0.565	*0.861*	0.647	0.377	0.579
CAC—Item 5	0.537	*0.853*	0.690	0.332	0.595
CAC—Item 6	0.544	*0.801*	0.647	0.338	0.566
KOC—Item 1	0.613	0.710	*0.856*	0.332	0.655
KOC—Item 2	0.485	0.552	*0.733*	0.378	0.604
KOC—Item 3	0.541	0.595	*0.804*	0.288	0.620
KOC—Item 4	0.499	0.525	*0.738*	0.368	0.619
KOC—Item 5	0.532	0.627	*0.828*	0.316	0.594
KOC—Item 6	0.583	0.634	*0.866*	0.366	0.661
KOC—Item 7	0.558	0.652	*0.874*	0.325	0.661
KOC—Item 8	0.515	0.617	*0.811*	0.278	0.546
KOC—Item 9	0.504	0.614	*0.844*	0.363	0.641
KOC—Item 10	0.441	0.548	*0.750*	0.340	0.616
PRK—Item 1	0.345	0.366	0.401	*0.815*	0.372
PRK—Item 2	0.249	0.276	0.301	*0.801*	0.343
PRK—Item 3	0.220	0.230	0.235	*0.838*	0.288
PRK—Item 4	0.332	0.407	0.380	*0.812*	0.434
PRB—Item 1	0.503	0.616	0.745	0.343	*0.789*
PRB—Item 2	0.435	0.474	0.587	0.330	*0.752*
PRB—Item 3	0.496	0.556	0.629	0.349	*0.808*
PRB—Item 4	0.501	0.565	0.614	0.281	*0.794*
PRB—Item 5	0.375	0.382	0.463	0.342	*0.666*
PRB—Item 6	0.430	0.470	0.522	0.306	*0.754*
PRB—Item 7	0.529	0.572	0.651	0.290	*0.777*
PRB—Item 8	0.528	0.577	0.634	0.327	*0.793*
PRB—Item 9	0.455	0.557	0.588	0.354	*0.783*
PRB—Item 10	0.449	0.528	0.551	0.375	*0.784*
PRB—Item 11	0.240	0.280	0.330	0.430	*0.540*
PRB—Item 12	0.315	0.375	0.420	0.399	*0.666*
**Fornell-Larcker criterion**					
APM	0.812				
CAC	0.750	0.764			
KOC	0.651	0.630	0.731		
PRK	0.414	0.405	0.360	0.737	
PRB	0.768	0.679	0.405	0.658	0.513
**HTMT ratio**					
APM	–				
CAC	0.826	–			
KOC	0.713	0.718	–		
PRK	0.454	0.460	0.424	–	
PRB	0.807	0.745	0.658	0.513	–

APM, awareness of precaution measurement; CAC, concern about COVID-19; KOC, knowledge of COVID-19; PRK, perceived risk; PRB, preventative behavior.

Italic values are item loading.

### Path analysis

After achieving acceptable reliability and validity from the structural assessment of the model, the following measurement assessment was carried out to test the study hypotheses. The adjusted *r*^2^ value for the four exogenous constructs (APM, CAC, KOC, and PRK) on PRB elucidated 63.35% of the change in PRB. The predictive relevance (Q^2^) value for the part of the model was 0.345, indicating medium predictive relevance (33).

Table 11 presents the model standardized path values, t-values, and significance level. The path coefficient between APM and PRB (β = 0.515, t = 10.425, *p* = 0.000) displayed a significantly positive effect of APM on PRB. This result offers significant statistical support for H1. The path value for CAC and PRB (β = 0.173, t = 3.636, *p* = 0.000) was significantly positive, thus providing significant statistical support for H2. The path between KOC and PRB (β = 0.109, t = 0.109, *p* = 0.008) illustrated the significantly positive influence of KOC on PRB, hence delivering the substantiation to support H3. The path coefficient for PRK and PRB (β = 0.127, t = 4.755, *p* = 0.000) portrayed a significantly positive effect, thus signifying support for H4. Table 11 presents the path coefficient outcomes.

**Table 11:** Hypotheses testing.

**Hypothesis**		**Coefficient**	**t-values**	**Sig**.	***r^2^***	***f* ^2^**	**Q^2^**	**Decision**
H1	APM → PRB	0.515	10.423	0.000		0.275		*Supported*
H2	CAC → PRB	0.173	3.636	0.000		0.033		*Supported*
H3	KOC → PRB	0.109	2.430	0.008		0.017		*Supported*
H4	PRK → PRB	0.127	4.755	0.000	0.636	0.036	*0.345*	*Supported*

APM, awareness of precaution measurement; CAC, concern about COVID-19; KOC, knowledge of COVID-19; PRK, perceived risk; PRB, preventative behavior.

### Importance-performance matrix analysis

The outcomes of importance-performance matrix analysis (IPMA), as revealed in Table 12, display that the APM emerged as the most vital cause in performing PRB with scores (0.537; 87.426), while the second most decisive factor for performing PRB with scores (0.175; 83.344) is CAC. The third most important factor for performing PRB is KOC with scores (0.103, 78.768), whereas the fourth most important factor for the performance of PRB is PRK with scores (0.102; 70.993).

**Table 12:** Importance-performance matrix.

**Target construct**	**PRB**	
**Variables**	**Total effect**	**Performance**
APM	0.537	87.426
CAC	0.175	83.344
KOC	0.103	78.768
PRK	0.102	70.993

APM, awareness of precaution measurement; CAC, concern about COVID-19; KOC, knowledge of COVID-19; PRK, perceived risk; PRB, preventative behavior.

### Multiple group analysis

The multiple group analysis (MGA) was performed to compare the results for different groups based on age and gender. A non-parametric test was employed to appraise the variances in the vital association between the models based on the age and gender of the respondents. Table 13 depicts the path values for two groups and the differences within the groups with *p*-values, as recommended by Henseler et al. (33). The P_MGA_ represents the *p*-values achieved *via* MGA of PLS-SEM as the measure of the significance of the difference between the groups under study (33).

**Table 13:** Multiple group comparison based on age.

	**Young**			**Old**			**Difference**	**P_MGA_**
	**β**	**t-values**	**Sig**.	**B**	**t-values**	**Sig**.		
APM → PRB	0.584	4.941	0.000	0.476	7.925	0.000	−0.108	0.211
CAC → PRB	0.400	2.307	0.011	0.114	2.366	0.009	−0.286	0.078
KOC → PRB	−0.100	0.758	0.224	0.159	3.249	0.001	0.259	0.040
PRK → PRB	0.033	0.543	0.294	0.153	4.093	0.000	0.120	0.037

APM, awareness of precaution measurement; CAC, concern about COVID-19; KOC, knowledge of COVID-19; PRK, perceived risk; PRB, preventative behavior.

Based on Table 13, groups based on age displayed significant differences in the correlations of PRB with KOC and PRK. The variance of age, nevertheless, had no impact on the relationships of PRB with APM and CAC.

The results of the two groups based on gender exhibited no significant difference in the relationships of PRB with APM, CAC, KOC, and PRK (see Table 14).

**Table 14:** Multiple group comparison based on gender.

	**Male**			**Female**			**Difference**	**P_MGA_**
	**B**	**t-values**	**Sig**.	**B**	**t-values**	**Sig**.		
APM → PRB	0.592	7.656	0.000	0.455	7.286	0.000	0.137	0.084
CAC → PRB	0.123	1.090	0.138	0.200	4.006	0.000	−0.076	0.277
KOC → PRB	0.134	1.420	0.078	0.111	2.134	0.017	0.023	0.407
PRK → PRB	0.132	2.722	0.003	0.116	3.579	0.000	0.015	0.412

APM, awareness of precaution measurement; CAC, concern about COVID-19; KOC, knowledge of COVID-19; PRK, perceived risk; PRB, preventative behavior.

## Discussion

The four hypotheses formulated in this study had assessed the effect of APM, CAC, KOC, and PRK on PRB among the study respondents. As a result, APM (*f*^2^ =0.275) displayed a positive and significant medium effect on PRB (20). The study results are in agreement with those reported by Choi and Kim (2), Alzaatreh et al. (16), and Teo et al. (34) that signified the significance of APM for preventing behavior among the study sample. Next, the effect of CAC (*f*^2^= 0.033) on PRB was positive and significantly medium (31), which is in line with the results reported by Almutairi et al. (7). The influence of KOC (*f*^2^= 0.017) was significantly small on PRB, which is supported by the results postulated by Choi and Kim (2), is inconsistent with Zhong et al. (35) that KOC has a significant negative effect on PRB. Lastly, the impact of PRK (*f*^2^= 0.036) on PRB was significantly small among the study respondents, which matched with the results reported by Ibuka et al. (19) and Aghababaei et al. (21).

In addition, this study explored the performance of PRB against COVID-19 with factors of APM, CAC, KOC, and PRK. The most critical factor that dictated the performance of PRB was APM, which supported the outcome research of Yildirim and Güler (36). The second vital factor was CAC, whereas the third and fourth essential factors for the performance of PRB were KOC and PRK. These results align with Alzaatreh et al. (16), which indicated that threat awareness would be the pathway through which knowledge and awareness about COVID-19, or any other disease or infectious disease, can influence adherence to preventive behaviors. Notably, improving APM in combatting COVID-19 emerged as the most crucial factor in achieving the required PRB. Low PRK was the most significant factor contributing to low PRB, similar to Girma et al. (37) and Dryhurst et al. (38). Essentially, electronic and social media should build awareness of the risk associated with COVID-19, highlighting the role of APM in significantly building PRB for COVID-19, since social media was a primary source for COVID-19 information (39).

This study explored the moderating effects of age and gender on the relationships of PRB with APM, CAC, KOC, and PRK. In light of the respondents' age, a significant variance was noted between young and old respondents for KOC and PRK with PRB. Both KOC and PRK reflected higher scores among the older respondents than the young respondents. The result is associated with the findings of Smail (22). The young people considered themselves less prone to COVID-19 and less inclined to engage in COVID-19 preventative practices. Nonetheless, insignificant variances existed between young and old respondents for APM and CAC with PRB. Since the media has been generating awareness, both old and young respondents equally exhibited KOC and CAC in promoting preventative behavior. As for the effect of gender on the relationships of PRB with APM, CAC, KOC, and PRK, the gender difference appeared insignificant for PRB. This outcome signified that gender insignificantly facilitated the promotion of PRB in light of APM, CAC, KOC, and PRK for COVID-19. These results supported the finding of Superio (39) that respondents' demographics did not significantly affect their precautionary actions during the pandemic outbreak. Contradicting these findings, Ning et al. (3) stated that women and older people were more likely to embrace protective behaviors than their male and younger counterparts. Soltan (12) also proved the significant difference between the gender that female senior students have higher knowledge and practice preventive behaviors.

## Implication

The study outcomes significantly contribute to the existing drives to curtail the spread of COVID-19. Notably, APM was the most significant predictor of PRB for COVID-19, followed by PRK and CAC. The study results coincide with the earlier work described in (1, 2, 6, 14, 35, 37, 40) on other earlier infectious diseases, such as N1H1 and MERS-COV. It was found that enhancing awareness about infectious disease and promoting effective prevention measures with the actual allied level of risk facilitated the mass adoption of PRB toward COVID-19. Moreover, the interactions among PRK, APM, and KOC strongly encouraged more individuals to display PRB in curbing the spread of COVID-19.

Policymakers may draw some guidelines from this study. Since a large fraction of the world reside in rural areas and have high-level interaction with animals, the provision of education at all level can harness the attitude to adopt PRB (11). Besides, it is integral for policymakers to work with the young generation so that the latter may serve as change agents to spread the message of taking precautions and adopting PRB (10). Including the topic of viruses and the promotion of PRB in primary education, the curriculum is pertinent. Updating the education curriculum may enable the world population to attain better healthy living and wellbeing in the near future as the spread of viruses has been rampant in recent years. As Superio (39) stated, to assist students in reducing fear of getting such information, it is necessary to implement programs that provide information literacy, helping them to tell the difference between true and false information. Schools should provide crisis-oriented programs to support students' psychological wellbeing (40).

## Conclusion

The rising COVID-19 pandemic across the globe demands global collaboration to instill personal preventive measures, which tend to be the only viable strategy available. The change in human lifestyle and animal-based infectious diseases calls for continuous global sustainable surveillance to identify and control the pandemic from reaching a severe level. The present COVID-19 pandemic involves the same breed of coronavirus found in animals, thus causing severe threats to human life worldwide.

Along with its strengths, this study has five shortcomings. First, the data gathered from a single source in a cross-sectional manner are associated with a lack of generalization. The longitudinal research design may generate more informative data and multiple sources. Second, the limited personal factors of age and gender could lead to potential variances among the study respondents. The specific attitudinal differences can significantly influence the adoption of PRB. Like mindfulness, hope and concern for public health can facilitate the exploration of individual variances to unearth PRB. Third, it would be worthwhile to capture data from the low-educated and more diversified segments to paint a clearer picture of the role of awareness in adopting PRB, particularly in the attempt to minimize the harmful impact of infectious diseases that may turn into a life-threatening pandemic. The fourth limitation of this work is related to the method of data collection and the Likert scale applied to gather responses from the respondents. Although an online survey offers the flexibility to both the researcher and the respondents to respond to the research questions, any essential first-hand information or in-depth discussion is lost in an online survey using the Likert scale. As such, future research should incorporate personal interviews and open questions to thoroughly explore the phenomenon investigated in this study. Finally, this study gathered data from university students who received plenty of COVID-related information from their universities. They were active in social media and most likely received massive amounts of relevant details from the platform. Both Malaysian and Vietnamese governments, together with various news agencies, should constantly broadcast imminent updates. It is more likely that university students in both countries are more aware of the situation and the potential consequences than the general population. The statically significant correlations of APM, CAC, KOC, and PRK with PRB may not reflect the general population of the two studied countries.

## Author's note

Online data collection was performed following the CHERRIES framework. The survey design was described, and approval was taken from the university level review board. Informed consent was taken from the respondents about the data protection. Pre-testing was performed during the survey development. The survey remained the open survey, and the contact information was received from the online survey platform (Google forms). The participation in the online survey was voluntary, and no incentive was offered during the online data collection.

## Data availability statement

The original contributions presented in the study are included in the article/Supplementary material, further inquiries can be directed to the corresponding author.

## Ethics statement

Ethical review and approval was not required for the study on human participants in accordance with the local legislation and institutional requirements. The patients/participants provided their written informed consent to participate in this study.

## Author contributions

AA contributed to the conception, research design, analyze and interpretation of data, and prepared the final draft. HD and NZ contributed to the research design, questionnaire design, and collected data from Vietnam and Malaysia respectively. NH and AS contributed to the conception, design of the work, and have drafted the manuscript. All authors have read and approved the manuscript.

## Conflict of interest

The authors declare that the research was conducted in the absence of any commercial or financial relationships that could be construed as a potential conflict of interest.

## Publisher's note

All claims expressed in this article are solely those of the authors and do not necessarily represent those of their affiliated organizations, or those of the publisher, the editors and the reviewers. Any product that may be evaluated in this article, or claim that may be made by its manufacturer, is not guaranteed or endorsed by the publisher.

## Abbreviations

APM: awareness of precaution measures
COVID-19: coronavirus disease 2019
CAC: concern about COVID-19
KOC: knowledge of COVID-19
PRK: perceived risk
PBR: preventive behavior
PLS-SEM: partial least square structural equation modeling
SEM: structural equation modeling
MGA: multiple group analysis
SARS: severe acute respiratory syndrome
MERS-COV: Middle East respiratory syndrome coronavirus
CMV: common method variance
AVE: average variance extracted
HTMT: heterotrait–monotrait
VIF: variance inflation factor.
